# Supplementary material for: Social organization of a solitary carnivore: spatial behaviour, interactions and relatedness in the slender mongoose
Source: R Soc Open Sci. 2019 May 8;6(5):182160. doi: 10.1098/rsos.182160 (PMC6549956; doi:10.1098/rsos.182160)
Supplement: Individual tracking data and range sizes [file rsos182160supp1.docx]

**Supplemental Material**

**Table A**: Tracking data and sleeping range sizes (n: total number of sleeping sites, months: number of months tracked, range sizes are given in km^2^).

|  | **n** | **months** | **overall range** | **2008-2009** | **2009-2010** | **2010-2011** |
| --- | --- | --- | --- | --- | --- | --- |
| SBF01 | 157 | 39 | 1.69 | 1.69 |  |  |
| SBF09 | 148 | 26 | 1.10 |  | 1.10 | 1.00 |
| SCF02 | 222 | 50 | 1.17 | 1.42 | 0.88 |  |
| SCF09 | 133 | 21 | 0.82 |  |  | 0.82 |
| SDF04 | 391 | 76 | 2.06 | 2.29 | 1.52 | 0.46 |
| SDF13 | 109 | 18 | 0.55 |  |  | 0.55 |
| SGF02 | 354 | 69 | 0.89 | 1.96 | 0.84 | 0.76 |
| SGF10 | 126 | 37 | 0.53 | 0.94 | 0.31 |  |
| SLF02 | 96 | 10 | 0.67 |  |  | 0.67 |
| SMF01 | 412 | 72 | 1.58 | 1.73 | 1.79 | 1.47 |
| SMF04 | 401 | 60 | 1.59 | 1.04 | 1.42 | 1.13 |
| SMF09 | 238 | 46 | 1.64 |  | 1.65 | 1.24 |
| SAM03 | 71 | 23 | 6.21 | 6.21 |  |  |
| SBM08 | 102 | 50 | 6.18 |  | 6.18 |  |
| SBM13 | 53 | 30 | 5.45 |  |  | 5.45 |
| SCM01 | 132 | 50 | 3.08 | 3.08 |  |  |
| SCM04 | 163 | 31 | 1.91 |  |  | 1.91 |
| SDM02 | 117 | 31 | 6.18 | 7.46 | 5.93 |  |
| SDM07 | 51 | 23 | 3.80 |  |  | 3.80 |
| SDM08 | 84 | 25 | 1.00 |  | 1.27 | 0.51 |
| SFM02 | 86 | 27 | 1.49 | 1.49 |  |  |
| SGM01 | 265 | 98 | 2.13 | 2.56 | 1.80 | 1.40 |
| SGM06 | 361 | 83 | 1.61 | 1.52 | 1.98 | 1.46 |
| SMM03 | 407 | 93 | 2.00 | 1.75 | 2.31 | 2.55 |
| SMM05 | 346 | 124 | 3.93 | 4.98 | 3.95 | 2.55 |
| SRM01 | 82 | 41 | 5.71 | 5.71 |  |  |
