# Supplementary material for: Social organization of a solitary carnivore: spatial behaviour, interactions and relatedness in the slender mongoose
Source: R Soc Open Sci. 2019 May 8;6(5):182160. doi: 10.1098/rsos.182160 (PMC6549956; doi:10.1098/rsos.182160)
Supplement: Individual site fidelity [file rsos182160supp2.docx]

**Supplemental Material**

| **1. 95% range size & overlap** | **2008-2009** | **2009-2010** | **2010-2011** | **overlap1** | **overlap2** |
| --- | --- | --- | --- | --- | --- |
| SBF09 |  | 1.10 | 1.00 |  | 82.9 |
| SDF04 | 2.29 | 1.52 |  | 57.6 |  |
| SGF02 |  | 0.84 | 0.76 |  | 76.9 |
| SMF01 | 1.73 | 1.79 | 1.47 | 91.7 | 78.2 |
| SMF04 | 1.04 | 1.42 |  | 65.2 |  |
| SMF09 |  | 1.65 | 1.24 |  | 60.4 |
| SGM01 |  | 1.80 | 1.40 |  | 58.0 |
| SGM06 | 1.52 | 1.98 | 1.46 | 90.3 | 71.3 |
| SMM03 | 1.75 | 2.31 | 2.55 | 98.6 | 94.0 |
| SMM05 | 4.69 | 3.95 | 3.99 | 88.8 | 87.1 |
|  |  |  |  |  |  |
|  |  |  |  |  |  |
| **2. 50% range size & overlap** | **2008-2009** | **2009-2010** | **2010-2011** | **overlap1** | **overlap2** |
| SBF09 |  | 0.30 | 0.18 |  | 42.5 |
| SDF04 | 0.57 | 0.36 |  | 50.0 |  |
| SGF02 |  | 0.13 | 0.13 |  | 56.1 |
| SMF01 | 0.42 | 0.45 | 0.38 | 68.9 | 58.7 |
| SMF04 | 0.23 | 0.37 |  | 59.8 |  |
| SMF09 |  | 0.44 | 0.33 |  | 37.1 |
| SGM01 |  | 0.38 | 0.27 |  | 67.5 |
| SGM06 | 0.36 | 0.36 | 0.27 | 86.6 | 63.6 |
| SMM03 | 0.52 | 0.52 | 0.50 | 68.0 | 66.4 |
| SMM05 | 1.47 | 0.99 | 1.10 | 60.7 | 62.0 |
|  |  |  |  |  |  |
|  |  |  |  |  |  |
| **3. % core area within 95%** | **overlap1** | **overlap2** |  |  |  |
| SBF09 |  | 98.3 |  |  |  |
| SDF04 | 100 |  |  |  |  |
| SGF02 |  | 100 |  |  |  |
| SMF01 | 100 | 100 |  |  |  |
| SMF04 | 100 |  |  |  |  |
| SMF09 |  | 70.8 |  |  |  |
| SGM01 |  | 100 |  |  |  |
| SGM06 | 100 | 100 |  |  |  |
| SMM03 | 100 | 100 |  |  |  |
| SMM05 | 100 | 100 |  |  |  |

**Table B**: Site fidelity for seven animals with at least 50 data points in at least two consecutive years; 1. 95% annual range size (in km^2)^ and overlap between years (overlap1: overlap between 2008-2009/2009-2010, overlap2: overlap between 2009-2010/2010-2011), 2. 50% (core area) range size and overlap between years (overlap1: overlap between 2008-2009/2009-2010, overlap2: overlap between 2009-2010/2010-2011), 3. % of core area within 95% range of following year.
