# Supplementary material for: Social organization of a solitary carnivore: spatial behaviour, interactions and relatedness in the slender mongoose
Source: R Soc Open Sci. 2019 May 8;6(5):182160. doi: 10.1098/rsos.182160 (PMC6549956; doi:10.1098/rsos.182160)
Supplement: Individual range drift [file rsos182160supp3.docx]

**Supplemental Material**

**Table C**: Range drift for ten slender mongooses based on shifts in range centers between consecutive years in meters (drift1: between 2008-2009 and 2009-2010; drift2: between 2009-2010 and 2010-2011; whole study: between first and last year tracked).

|  | **drift1** | **drift2** | **whole study** |
| --- | --- | --- | --- |
| SBF09 |  | 128 | 128 |
| SDF04 | 503 |  | 503 |
| SGF02 |  | 164 | 164 |
| SMF01 | 40 | 58 | 65 |
| SMF04 | 423 |  | 423 |
| SMF09 |  | 305 | 305 |
| SGM01 |  | 299 | 299 |
| SGM06 | 314 | 27 | 288 |
| SMM03 | 166 | 64 | 103 |
| SMM05 | 143 | 135 | 8 |
